# Genetic and cellular aspects of the establishment of histocompatible stem cells: information gained from an animal model

**DOI:** 10.1186/1753-6561-5-S4-S31

**Published:** 2011-06-03

**Authors:** Jeong Mook Lim, Seung Pyo Gong

**Affiliations:** 1 WCU Biomodulation Program, Seoul National University, Seoul 151-742, Korea; 2 Department of Agricultural Biotechnology, Seoul National University 151-921, Korea; 3 Department of Marine Biomaterials and Aquaculture, College of Fisheries Science, Pukyong University, Busan 608-737, Korea

## Abstract

The establishment of patient-specific histocompatible stem cells may be an alternative for overcoming current limitations in stem cell engineering. We are developing an animal model to assist the establishment of histocompatible, autologous stem cells. In this process, we obtained valuable information on establishing and characterizing stem cells. As an initial step, we succeeded in establishing histocompatible stem cells using preantral follicle cultures and subsequent parthenogenetic activation. The gene expression profile of the established stem cells was similar to that of embryonic stem cells (ESCs) derived from normal fertilization. On the other hand, we propose a way to derive histocompatible, ESC-like cells by co-culturing ovarian stromal cells with feeder fibroblasts, which may allow the derivation of stem cells from somatic tissue. However, more progress regarding the establishment and elucidation on origination of established cell lines is necessary to use this genetic manipulation-free procedure. Nevertheless, relevant information on the process will help to stimulate preclinical research on cell transformation into differentiated, undifferentiated, and even cancerous cells, as well as clinical studies on the application of induced pluripotent cells.

## Background

There are several limitations to the use of stem cell engineering and stem cell-derived biomaterials for therapeutic purposes. The establishment of histocompatible or immune-specific stem cells for a patient is a prerequisite for developing stem cell engineering beyond its present limitations [1]. The development of an effective method for allowing differentiation into target cells would contribute greatly to facilitating stem cell engineering for cell therapy. Furthermore, the optimization of stem cell transplantation techniques and the development of a way to effectively regain normal function after stem cell transplantation are the final requirements for the cell therapy. From a different perspective, ethical, legal, and social considerations regarding stem cell technology will inevitably influence the development of clinically friendly stem cell engineering.

## Methods

We have focused on developing advanced technology for establishing histocompatible stem cells. A series of studies to develop novel stem cell engineering approaches arose from our failure to establish genetically stable embryonic stem cells (ESCs) upon the interspecies nuclear transfer of human somatic cells into bovine enucleated oocytes [2]. In fact, somatic cell nuclear transfer technology cannot guarantee complete cloning due to the presence of mitochondrial DNA [3], which causes phenotypic heterogeneity. In addition, there is a huge oocyte reservoir in the ovaries, with each ovary possessing approximately one million preantral (primordial, primary, and secondary stages) follicles. In various species, most preantral follicles are not mobilized to derive viable oocytes throughout the lifetime of the organism because of spontaneous degeneration. Given this situation, we considered the development of a technology that combines *in vitro* folliculogenesis and parthenogenesis to establish histocompatible stem cells.

As another approach, we attempted to derive histocompatible stem cells from terminally differentiated tissue. To derive the stem cells, we examined various cell niches involving feeder cells and the stromal cell population, which potentially includes stem cells or their progenitor cells. The success of this approach enormously expands the feasibility of stem cell engineering for both preclinical research and clinical application. In addition, valuable information on cell transformation into differentiated, undifferentiated, and even cancerous cells can be obtained from this cell transformation model.

## Results

### Information from follicle cultures and oocyte parthenogenesis

At any given time, numerous preantral follicles are present at different stages in mammalian ovaries. Throughout a woman’s life, less than 0.1% of immature follicles develop to the mature stage capable of yielding developmentally competent oocytes [4]. The others either disappear after birth or remain in a ‘developmentally dormant’ state in the ovarian cortex or medullary tissues [5]. Theoretically, many immature follicles can be used to derive developmentally competent oocytes, which could be linked to establish histocompatible (autologous) stem cells following parthenogenetic activation. Using this strategy, the abuse of viable oocytes derived from normal folliculogenesis and natural ovulation can be avoided, although the cloning of oocyte donors cannot be completely eliminated. We subsequently elaborated on the culturing of preantral follicles for deriving developmentally competent oocytes.

As the first step in establishing a follicle culture, we used neonatal mice as follicle donors, in order to retrieve a large number of preantral follicles [6]. The follicles were retrieved mechanically, without enzyme treatment, to prevent the cellular damage caused by enzyme treatment and thus improve follicle development [7-9]. The retrieved follicles were washed three times and cultured singly in 20-μl culture droplets [7,10]. Of the three stages of immature follicles, we used primary or early secondary follicles for *in vitro* culture. Either α-minimal essential medium or Dulbecco’s modified Eagle’s medium (DMEM) was used as the basal medium [6,7,10-13], and serum, growth factors, antibiotics, and other substances were added. In particular, various amounts of follicle-stimulating hormone and luteinizing hormone were added to the culture medium because of their importance in follicle growth and oocyte maturation [13-17]. The duration of *in vitro* culture is an important factor for enabling the full development of preantral follicles. Primary follicles were cultured for 11 days, and early secondary follicles were cultured for 9 days [7,10]. For the parthenogenetic activation of oocytes derived from the follicle culture, we selected a treatment using Ca^2+^-free KSOM medium supplemented with SrCl_2_ and cytochalasin B. To derive colony-forming cells from parthenogenetic blastocysts, inner cell mass (ICM) cells of a blastocyst attaching to the feeder cell were isolated and subcultured. When the attached ICM cells were stably maintained as colony-forming cells, their stem cell nature was evaluated using a variety of markers, assays, and differentiation techniques.

**Figure 1:**
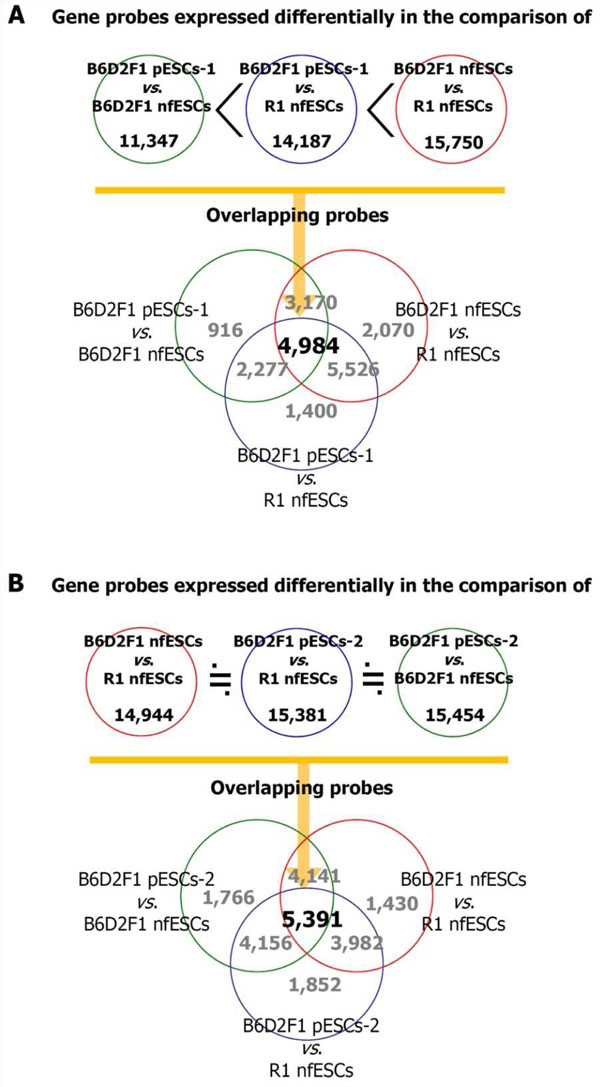
**Comparison of the gene expression profiles of embryonic stem cells (ESCs) derived from different origins**. Two sets (A and B) of comparisons were made using two lines of parthenogenetic ESCs (pESC-1 and pESC-2 for A and B, respectively). In each set, the gene expression profile of normally fertilized ESCs (nfESCs) derived from R1 strain was first compared with the profile of nfESCs derived from B6D2F1 strain. Comparisons were subsequently made between pESCs and nfESCs of the same strain (B6D2F1) and between pESCs and nfESCs of a different strain (R1). In the first comparison (A), the change in gene expression after parthenogenesis was less than the change attributable to the strain difference; the number of genes with altered expression was similar among all comparisons in the second set (B). (Reprinted with permission from Gong *et al*., Hum Reprod 2009; 24: 815-814).

A number of stem cell lines have been derived from follicle cultures, and the original system has been optimized by adding or deleting specific factors. To assess the clinical usefulness of our technology, it was important to determine whether the parthenogenetic stem cells had a potential for self-renewal and differentiation as well as physiological and genetic properties that were similar to those of normally fertilized stem cells or reported ESCs. The gene expression profiles of two parthenogenetic ESC lines were compared with those of two normally fertilized ESC lines using genome-wide microarray analysis [18]. The mRNA expression levels were quantified to validate the data. As shown in Fig 1, in two comparisons, i.e., one for each parthenogenetic ESC line, the reactions of 11,347 and 15,454 gene probes, respectively, were altered by parthenogenesis, while strain differences accounted for changes in the reactions of 15,750 and 14,944 probes. The correlation coefficient was higher for the comparisons between normally fertilized and parthenogenetic ESCs than for the comparisons between strains of normally fertilized ESCs. The expression levels of approximately 3,300 genes were changed after parthenogenesis, and about 90% of the major functional genes differentially expressed in one comparison set showed the same difference in the other comparison. The expression levels of several paternally and maternally imprinted genes were also altered after parthenogenesis, and the differentially expressed genes were similar for each comparison. Thus, although altered expression of some genes was observed in ESCs after parthenogenesis, the degree of alteration was similar to or less than that observed between ESC strains of the same derivation in a given genetic environment. Further information on cell differentiation and genetic stability *in vivo* after transplantation, as well as *in vitro*, is needed to confirm the clinical feasibility of our system.

The establishment of supportive programs will contribute to increasing the clinical applicability of the follicle culture system in reproductive biotechnology and infertility medicine, as well as in stem cell engineering. To date, we have succeeded in deriving developmentally competent oocytes by culturing preantral follicles retrieved from the ovaries of adults [19] and the elderly [20]. The culture of primary follicles can produce developmentally competent oocytes [21]. An ovarian tissue bank has been established using an optimized cryopreservation protocol [22, 23].

### Information from cultures of different cell types for deriving histocompatible stem cells

In another approach, we endeavored to derive autologous stem cells by culturing somatic cells. To induce cell transformation into stem cells, we co-cultured ovarian stromal cells and fetal fibroblasts. This was suggested by a recent report on the presence of tissue-specific stem cells in non-germline ovarian cells [24]. Germline stem cells have also been identified in adult ovaries [25, 26]. Ovarian stromal cells include blood and blood vessel cells, connective tissue cells consisting of mesenchymal cells, nerve cells, secreting cells, and primordial germ cells, and it is highly possible that various types of mesenchymal cells are mixed with fetal fibroblasts. Although only a crude design was proposed, the co-culture of ovarian stromal cells with embryonic fibroblasts increased the possibility of inducing stem cell transformation by cell-to-cell interactions among co-existing cells. In fact, stem cells and their extracts have been shown to stimulate the transformation of terminally differentiated cells into stem cells [27, 28]. By utilization of cell-to-cell interaction, we did not employ genetic manipulation for stem cell establishment in order to increase the clinical practicality of the proposed cell transformation system.

We first surveyed the expression of stemness-related genes in individual ovaries of different animals. Every ovary expressed *Oct-4*, *Cripto*, *Rex-1*, *Dnmt3b*, *Tert*, and *Lif R*, and all but one also expressed *Nanog*. However, fetal fibroblasts did not show strong signals for stemness-related gene expression, demonstrating that an extremely small number of the cells expressing stemness-related genes are present among fetal fibroblasts. Consequently, ovarian stromal cells were pre-filtered with a cell strainer to dissociate ovarian cells less than 40 μm in diameter, in order to eliminate growing oocytes and most primary and early secondary follicles from a mixed cell population. The dissociated cells were co-cultured with fetal fibroblasts in DMEM-based medium, and two lines of colony-forming cells were derived from the culture [29]. These colony-forming cells were morphologically similar to reported ESCs. Both colony-forming cell lines were continuously maintained in culture and remained positive for stem cell-specific markers; stem cell-associated genes were expressed in the cell lines, with a high level of telomerase activity. Both lines had a normal diploid karyotype, with XX sex chromosomes. Markers of germline or ovarian follicular cells were not detectable in these cell lines. After culture in leukemia inhibitory factor-free medium, the established cells formed embryoid bodies. After subcutaneous transplantation into NOD-SCID mice, the injected cells formed teratomas consisting of cells derived from the three germ layers; however, they failed to induce germline transmission in a limited number of attempts.

**Figure 2:**
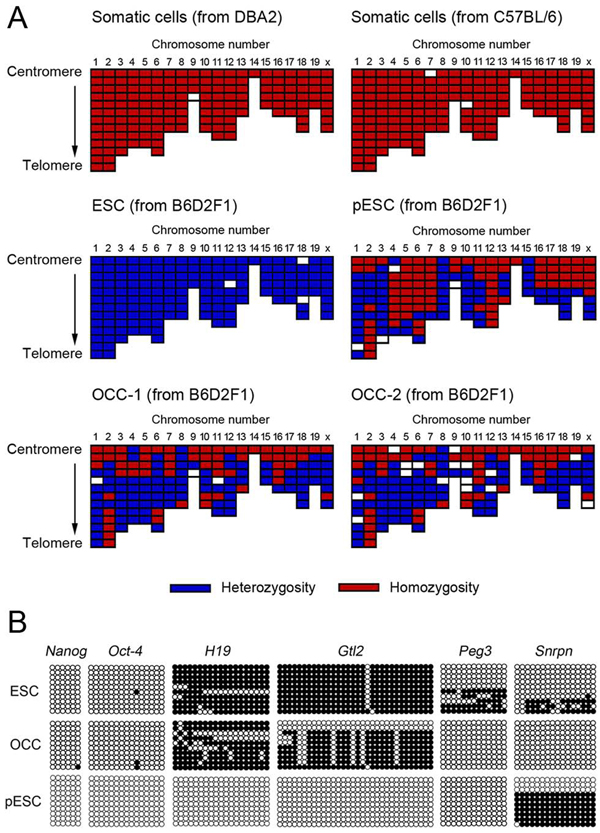
**Analyses of the origin of ovarian colony-forming cells (OCCs).** (A) Single nucleotide polymorphism (SNP) genotyping of OCCs and control cells. The heterozygosity or homozygosity of SNP loci of OCC-1 and OCC-2 of B6D2F1 strain was compared with that of B6D2F1 embryonic stem cells (ESCs), somatic fibroblasts of DBA2 and C57BL6 mice, and parthenogenetic ESCs (pESCs). Both homozygosity and heterozygosity were concomitantly detected in the OCC line. ESCs of F1 strain showed heterozygosity alone, and only homozygotic SNP loci were detected in the fibroblasts of the inbred strain. The pESC line possessed both homozygotic and heterozygotic chromosomes. (B). Methylation status of OCCs, ESCs, and pESCs. Genomic DNA isolated from these cells was subjected to bisulfite genomic sequencing analysis. The methylation levels of the promoter regions of stemness-related genes (*Oct-4* and *Nanog*) and imprinted genes expressed differentially after parthenogenetic activation (*H19*, *Peg3*, *Snrpn*, and *Gtl2*) were compared. The PCR products were cloned, and 10 plasmid clones were sequenced for each sample. Open and closed circles indicate unmethylated and methylated CpG dinucleotides, respectively. Stemness-related genes were demethylated in all cell lines, whereas the expression of other genes differed markedly among the cell lines. The methylation in OCCs was significantly different from that in ESCs or pESCs; OCCs had more methylated *H19* and *Gtl2* compared with pESCs and less methylated *Peg3* and *Snrpn* compared with ESCs. (Reprinted with permission from Gong *et al.*, 2010; 93:2564-601).

To further understand the origin and genetic characteristics of the two colony-forming cell lines, we conducted a short tandem repeat microsatellite analysis, which showed that the two colony-forming cell lines were an identical match to the ovary donor. Furthermore, we performed single nucleotide polymorphism genotyping and methylation analysis of the cell lines, to determine their origin. Homozygotic and heterozygotic chromosome recombinations were detected in both the colony-forming cell lines and the parthenogenetic ESC lines. The ESCs of the F1 strain possessed only heterozygotic loci, whereas homozygotic recombination was detected in the fibroblasts of both inbred strains. In the methylation analysis, No difference in the methylation of stem cell-related genes (*Nanog* and *Oct-4*) was seen among the parthenogenetic ESCs, normally fertilized ESCs, and ovarian colony-forming cells, although there was a significant difference in the methylation of several imprinted genes between ovarian colony-forming cells and parthenogenetic ESCs (Fig. 2). Based on these results, we have identified and characterized a novel colony-forming cell population that appears to be derived from either cell transformation accompanying somatic cell reprogramming or from tissue-specific stem cell-progenitor cell isolation. Nevertheless, we cannot completely exclude the tumorigenesis of ovarian somatic cells or parthenogenetic cells as possible origins. Further research is necessary to obtain conclusive results regarding cell origin and to evaluate the feasibility of clinical and industrial applications.

## Conclusion

We succeeded in deriving autologous stem cells by culturing primary or early secondary follicles followed by oocyte parthenogenesis. The genetic characteristics of the follicle culture-derived stem cells produced by parthenogenetic activation were similar to those of ESCs produced by normal fertilization. The follicle culture technique can be used to obtain developmentally competent oocytes from degeneration-fated, preantral follicles, for reproductive purposes. The successful derivation of histocompatible stem cells from the co-culture of ovarian tissue cells and fetal fibroblasts will have a great impact on the generation of patient-specific cells from stem cells. Further effort, however, is necessary to confirm the feasibility of producing colony-forming cells and to elucidate the origin of the established cells.

## Lists of abbreviations used

ESC(s): Embryonic Stem Cell(s); ART: Artificial Reproductive Technology; ICM: Inner Cell Mass; DMEM: Dulbecco’s Modified Eagle's Medium; α-MEM: α-minimal essential medium

## Competing interests

The authors declare that they have no competing interests.
